# Impact of Pneumococcal Conjugate Vaccines on Pneumonia Hospitalizations in High- and Low-Income Subpopulations in Brazil

**DOI:** 10.1093/cid/cix638

**Published:** 2017-07-22

**Authors:** Joshua L Warren, Kayoko Shioda, Esra Kürüm, Cynthia Schuck-Paim, Roger Lustig, Robert J Taylor, Lone Simonsen, Daniel M Weinberger

**Affiliations:** 1 Department of Biostatistics; 2 Department of Epidemiology of Microbial Diseases, Yale School of Public Health, New Haven, Connecticut; 3 Department of Statistics, University of California, Riverside; 4 Sage Analytica, Portland, Maine; 5 Department of Public Health, University of Copenhagen, Denmark

**Keywords:** pneumococcal conjugate vaccines, disparities, pneumonia, Brazil

## Abstract

**Background:**

Pneumococcal conjugate vaccines (PCVs) are being used worldwide. A key question is whether the impact of PCVs on pneumonia is similar in low- and high-income populations. However, most low-income countries, where the burden of disease is greatest, lack reliable data that can be used to evaluate the impact. Data from middle-income countries that have both low- and high-income subpopulations can provide a proxy measure for the impact of the vaccine in low-income countries.

**Methods:**

We evaluated the impact of PCV10 on hospitalizations for all-cause pneumonia in Brazil, a middle-income country with localities that span a broad range of human development index (HDI) levels. We used complementary time series and spatiotemporal methods (synthetic controls and hierarchical Bayesian spatial regression) to test whether the decline in pneumonia hospitalizations associated with vaccine introduction varied across the socioeconomic spectrum.

**Results:**

We found that the declines in all-cause pneumonia hospitalizations in children and young and middle-aged adults did not vary substantially across low and high HDI subpopulations. Moreover, the estimated declines seen in infants and young adults were associated with higher levels of uptake of the vaccine at a local level.

**Conclusions:**

These results suggest that PCVs have an important impact on hospitalizations for all-cause pneumonia in both low- and high-income populations.

## Teaser

The results in this article suggest that PCVs have an important impact against hospitalizations for all-cause pneumonia in both low- and high-income populations.

Pneumococcal conjugate vaccines (PCVs) used in children have had a well-documented impact on invasive pneumococcal disease [1] and pneumonia [2, 3] in high- and middle-income countries where they have been used since 2000. More recently, PCVs have been introduced in many low-income countries around the world [4]. However, few data are available that allow for comparison of the impact of PCVs on pneumonia between low- and high-income settings.

The impact of PCVs is likely to differ between high- and low-income settings for many reasons. For example, differences in immune and nutritional status, intensity of transmission, and the frequency of various pneumococcal strains [5] and other respiratory pathogens could all lead to a greater or smaller reduction in all-cause pneumonia following the introduction of PCVs. Unfortunately, despite this potential for differences in the impact of PCVs between settings, data needed to monitor changes in disease rates are usually not available in low-income settings.

Data from middle-income countries, which contain relatively poor and affluent subpopulations, can provide a comparison of vaccine impact between low- and high- income populations. Brazil, which introduced PCV10 in 2010 using a 3 + 1 dosing schedule for infants (doses at 2, 4, 6, and 12 months of age) and a catch-up dose for children aged 12–23 months, is one such country. We therefore applied complementary time series analyses and spatiotemporal analyses to assess PCV-associated declines in all-cause pneumonia hospitalizations in low- and high-income subpopulations of Brazil.

## METHODS

### Hospitalization Data

In Brazil, comprehensive data are available on individuals who receive publicly funded healthcare; these accounted for approximately 82% of the national population in 2012, including >2.5 million infants aged <12 months, although the proportion covered varied locally [6]. We obtained deidentified, age-stratified, *International Classification of Diseases, Tenth Revision, Clinical Modification* (ICD-10), coded data on hospitalizations (January 2004–December 2013) from the Unified Health System (SIH-SUS, Ministry of Health). “All-cause pneumonia” was defined as the occurrence of the ICD10 codes J12–J18 in a patient’s diagnosis field. Individuals with certain treatment packages codes (eg, post-transplant complications) were excluded to reduce the number of cases of hospital-acquired pneumonia. The control variables were defined as in Bruhn et al [7]. The database, data quality issues, and case definitions have been described in detail previously [6, 7].

### Geographic Classifications

Brazil is divided into 5570 municipalities, 136 mesoregions (groupings of municipalities that are smaller than states), 27 states, and 5 geographic regions. The 5 regions of Brazil correspond roughly to different climatic conditions (defined by the Brazilian Institute of Geography and Statistics as north, northeast, center-west, southeast, and south).

For the synthetic control (SC) analyses, the monthly hospitalization data from the municipalities were aggregated by region and by 3 HDI levels [8]. The development categories were based on the HDI for 2010 and grouped by very low/low development (<0.60), medium development (0.60 to <0.70), or high/very high development (≥0.70). The HDI summarizes the condition of a locality based on life expectancy, education level, and gross national income per capita, with higher values indicating improved living conditions. For the spatiotemporal disease mapping model, all analyses were performed at the mesoregion and yearly aggregation levels, with each mesoregion likewise classified by development and by region.

### Pneumococcal Conjugate Vaccine Coverage Data

The PCV coverage data were obtained from an administrative database (National Immunization Program) that tracks the number of first, second, third, and booster doses administered for each age group in each municipality and year [9]. We found that data quality at the municipality level was poor due to mismatches in the numerator (number of children vaccinated in a municipality, regardless of where they live) and denominator (based on number of children born in a municipality), which often resulted in vaccine uptake estimates >100%. Therefore, we aggregated the data to the mesoregion level. The denominator for vaccine uptake was defined as the number of children in the respective age band and was estimated from municipality-specific live-birth statistics by month (Sistema de Informações sobre Nascidos Vivos, Brazilian Ministry of Health). A cohort model (described in detail previously [10]) was used to estimate the percentage of children aged 6–24 months who received 3+ doses of vaccine or who received the age-appropriate vaccine (eg, only 1 catch-up dose after age 1 year for children not age eligible for the full series).

### Time Series Analysis Using Synthetic Controls

Our statistical analyses tested whether pneumonia hospitalizations declined over time in the post-PCV10 period. To adjust for trends unrelated to the vaccine (eg, due to changes in healthcare delivery and use [6], changing demography, or other nonspecific health interventions), we used the SC method, with the same variables and prior specifications as in our previous analyses of national-level data from Brazil [7, 11]. The SC method first establishes an association between pneumonia and control diseases using time series data from the prevaccine period to create a composite, “synthetic” control. It then uses the behavior of the composite control time series in the post-vaccine period to adjust for trends unrelated to the vaccine. The 2 major assumptions are that the vaccine does not influence the control time series and that the association between pneumonia and the control time series is consistent over time and is only influenced by the vaccine. The models were fit to the pre-PCV10 data (2004–2009) and used to generate a counterfactual estimate of the number of pneumonia hospitalization cases that would have occurred without vaccine introduction for the period 2012–2013. The rate ratios (RRs) were calculated by comparing the total number of observed cases and counterfactual cases for 2012–2013. The analysis was performed separately for national-level data and for data disaggregated by region or by HDI level. In sensitivity analyses, the top 1, 2, or 3 weighted variables were excluded, and the models were refit.

### Mesoregion-Level Spatiotemporal Model

We evaluated the association between pneumonia hospitalizations and uptake of PCV10 using a varying coefficient Poisson regression model that explicitly models spatial relationships among mesoregions [12, 13]. The outcome was the number of hospitalizations for all-cause pneumonia in a given year and mesoregion. The main predictor of interest was the uptake of PCV10 among children aged 6–23 months in that same year and mesoregion. We also included nonrespiratory hospitalizations as a covariate to adjust for variations in the volume of hospitalizations and population size, and we included other specific disease categories identified in the SC analysis as control variables (see Supplementary Materials). The model was structured to minimize confounding the effects of the vaccine with secular time trends, to control for unrelated spatially and temporally varying confounders, and to share information between related geographic areas and development levels during estimation of the parameters. For details on the model, see the Supplementary Materials.

As a check on the results, we also fit a standard negative binomial regression model where the outcome was hospitalization for pneumonia per year in a mesoregion and the covariates were nonrespiratory hospitalizations in that year and mesoregion (log scale), a dummy variable for year, a dummy variable for region, and vaccine uptake in the mesoregion and year.

The RR specific to each mesoregion and year was estimated by multiplying the estimated mesoregion-specific regression slope by the vaccine uptake for that mesoregion in a given year and exponentiating this quantity. This RR describes the expected change in all-cause pneumonia hospitalization counts when going from no vaccine to the level of uptake in the given year. Marginal mean estimates for the entire population and for each HDI group and region were calculated as described in the Supplementary Materials.

Inference for the spatiotemporal model was carried out in the Bayesian setting using the R-INLA (integrated nested Laplace approximation) package in the R statistical software program [14–16]. All parameters were assigned weak, minimally informative prior distributions (see Supplementary Materials).

### Spatiotemporal Model Sensitivity Analysis

In a sensitivity analysis, we randomly swapped the vaccine uptake time series among mesoregions within a region and reran the spatiotemporal statistical model to estimate the vaccine effects of interest. If the association between vaccine uptake and pneumonia is not confounded, estimates of the RR from models with swapped data should be centered around 1 (ie, no association). However, if vaccine uptake is actually confounded with a variable that is not currently included in our model, we would expect similar vaccine effect estimates as in the true (unswapped) analyses. Results that are similar between the 2 analyses for a particular age group suggest that the observed results may be due to confounding and should be interpreted with caution. To compare the estimates from the model with the observed or swapped data, we subtracted the swapped vaccine effect estimate from the true vaccine effect estimate for each mesoregion, exponentiated to convert to the RR scale, and plotted them as in the main analysis. For more details on the sensitivity analysis, see the Supplementary Materials.

## RESULTS

### Characteristics of the Databases

During the study period (2004–2013), hospital discharge data for Brazil included >2.9 million hospitalizations with a discharge code of “pneumonia” (J12–J18) among children aged <5 years living in 136 mesoregions (5570 municipalities) throughout the country. PCV10 was introduced in 2010. Uptake was high by the end of our study period (median uptake of 86% among children aged 6–23 months nationally in December 2013) but varied considerably by mesoregion, ranging from 50% to 97% (Supplementary Figures S1 and S2). A broad range of HDI values was represented among the municipalities, ranging from “very low” to “very high” development, although relatively few municipalities fell into the development categories at the extremes (Supplementary Figures S1 and S2).

### Changes in All-Cause Pneumonia Hospitalizations Do Not Vary by Development Level (Time Series)

**Figure 1.**
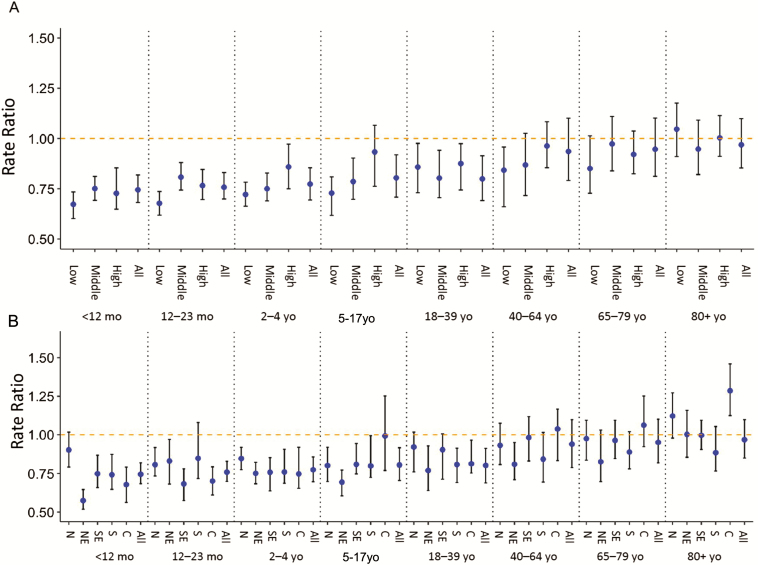
Change in pneumonia hospitalizations following the introduction of the 10-valent pneumococcal conjugate vaccine in Brazil as estimated with the synthetic control model, comparing the observed and predicted number of cases in 2012–2013 by age group and human development index level (A) and by age group and region (B). Median +/–95% credible intervals. Abbreviations: N, north; NE, northeast; C, center-west; SE, southeast; S, south.

First, we evaluated overall changes in the occurrence of pneumonia following the introduction of PCV10 in 2010 using the SC method (Figure 1, Supplementary Figures S2 and S3). Hospitalizations for all-cause pneumonia declined by 25.5% (95% credible interval [CI], 18.1%, 31.6%) nationally among children aged <12 months. When we further stratified the <12 month age group, we found a decline of 22% among infants aged <3 months (95% CI, 12%, 29%) and 23% (95% CI, 17%, 29%) among infants aged 3–11 months. Similar declines were estimated for older children and young adults, while no declines were detected using national data for adults aged 40–64, 65–79, or ≥80 years (Figure 1, Supplementary Figure S3).

The declines that occurred in children and young adults following the introduction of PCV10 were comparable in localities that were categorized as high/very high, medium, and very low/low development (Figure 1). In adults aged 40–64 years, declines in pneumonia were only detected in areas classified as very low/low development. No decline in all-cause pneumonia hospitalizations was detected in adults aged 65–79 and ≥80 years for any level of development (Figure 1). We performed sensitivity analyses in which the most heavily weighted control variables were dropped from the SC model and found that the results did not change appreciably (Supplementary Table S1). We also evaluated a simple model that adjusted only for seasonality and the volume of nonrespiratory hospitalization (Supplementary Figure S4).

### Changes in All-Cause Pneumonia Hospitalizations by Development Level (Spatiotemporal Analysis)

**Figure 2.**
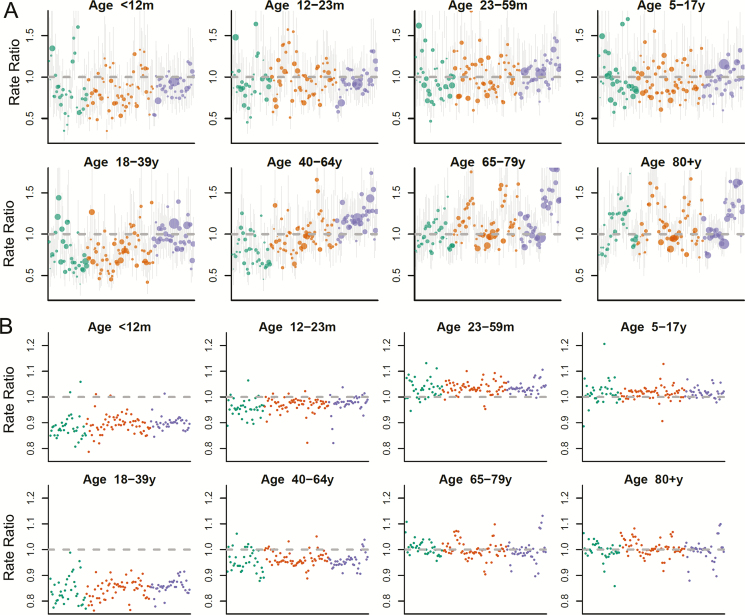
*A,* Rate ratio (RR) by mesoregion, with bubbles colored by human development index (HDI) level. The size of the bubble is proportional to the number of cases of pneumonia in that age group and mesoregion in 2013. *B,* Ratio of the RRs calculated using the observed coverage data and the median of the RRs calculated using swapped coverage estimates. A ratio of 1 indicates that there was no true effect of the vaccine. Colors denote HDI level: green = very low/low, orange = medium, purple = high/very high.

In a complementary analysis, we used a spatiotemporal model to determine whether changes in all-cause pneumonia hospitalizations across the 136 mesoregions in Brazil were associated with variations in the uptake of PCV10. We found that higher uptake of PCV10 in a mesoregion was associated with fewer hospitalizations for pneumonia among children aged <12 months (marginal mean RR, 0.83; 95% CI, 0.72, 0.96) and among adults aged 18–39 years (marginal mean RR, 0.81; 95% CI, 0.70, 0.93). The marginal mean represents the estimated decline for each age group associated with increasing vaccine coverage from zero to the national median uptake among infants in 2013 (86%), while controlling for development levels and region. The strength of this association did not vary significantly among very low/low-, medium-, and high/very high-development mesoregions (Figure 2). When we further stratified the youngest age group, we detected a significant association among children aged <3 months (as in the SC analysis). We did not detect an association between variations in uptake of PCV10 and pneumonia for children aged 3–11 months or for any of the other age groups (overall marginal mean RR estimates did not differ significantly from 1). The age pattern of estimates from a more standard regression model was similar to that of the spatiotemporal model—significant negative association in infants aged <12 months and those aged 18–39 year, a significant positive association in those aged ≥80 year (not seen in the spatiotemporal model), and no association for other age groups.

### Vaccine Impact Does Not Vary Appreciably by Mesoregion and Region

Estimates from the SC model (Figure 1B) and the spatiotemporal model (Supplementary Figure S5) suggested that there were variations in vaccine impact by mesoregion and region that could not be explained by variations in vaccine coverage. To explore potential biases that could influence the subnational estimates, we performed a sensitivity analysis for the spatiotemporal model in which we used vaccine uptake time series that had been randomly swapped among mesoregions within the same region. If the associations in the original model were confounded by a time-varying regional factor, we would expect that the swapped time series would show similar associations. Importantly, for children aged <12 months and young adults aged 18–39 years, the swapped results showed that the overall association between PCV10 and pneumonia was not confounded (Figure 2B). However, much of the variability between mesoregions could, in fact, be attributed to confounding. Notably, the apparent increase in cases associated with the vaccine in the older age groups was likely due to confounding, as was much of the variability between mesoregions in the other age groups (Figure 2B).

## DISCUSSION

The impact of PCVs on pneumonia hospitalizations in high-income settings has been well established [2]. However, few data are available on the population-level impact of PCVs on pneumonia in low-income countries [17–19]. Using subnational data from Brazil, we demonstrated that declines in the occurrence of hospitalizations for pneumonia in children and young to middle-aged adults following the introduction of PCV10 were consistent across regions classified as very-low/low, medium, and high/very-high development. This supports the notion that PCVs should reduce pneumonia hospitalization in both high- and low-income populations. While the low-income regions of Brazil are not a perfect proxy for low-income populations in other regions, the living conditions might be similar to those seen elsewhere (eg, large urban slums and isolated rural populations). Because the incidence of pneumonia and other pneumococcal diseases is so much greater in low-income countries than in high-income countries [20], even a modest benefit of the vaccine would translate into a large number of hospitalizations prevented.

The 2 complementary approaches that we used to evaluate the impact of PCV10 on hospitalizations for pneumonia yielded different estimates for certain age groups (eg, children aged 3–11 month). The time series analysis (SC method) measures the overall decline in pneumonia hospitalizations that occurs following vaccine introduction, while the spatiotemporal analysis assesses whether variability in pneumonia hospitalizations between localities can be attributed to higher uptake of PCV10. Because the 2 approaches measure different quantities, it is not surprising that they would yield different estimates. In particular, the spatiotemporal model controls for overall temporal declines in pneumonia common to all mesoregions using a dummy variable for year. As a result, the model effectively quantifies only the residual variation between mesoregions. Therefore, the lack of a significant effect in some age groups with the spatiotemporal model does not mean that PCV10 failed to reduce pneumonia hospitalizations in these age groups. Instead, it suggests that there simply is not a linear association over time between uptake of PCV10 and all-cause pneumonia hospitalizations at the mesoregion level and/or that there is not enough variability in uptake across time between the mesoregions to estimate a significant difference. Noise in the mesoregion-level data (eg, due to changes in child welfare programs) could potentially obscure a true association, as could inaccuracies in the vaccine uptake data. Additionally, vaccine uptake among children aged 6–23 months might not be the measure of vaccine coverage most relevant to the other age groups, and the effects might be nonlinear. Finally, if the indirect effect relies on a particular threshold of uptake being reached, then there might not be a clear dose–response linear relationship as the model assumes.

The analyses presented here have several notable strengths. We used a large database of hospitalizations for pneumonia and applied 2 complementary statistical approaches to estimate changes associated with PCV10 introduction. The SC approach is a robust method for reducing the influence of unmeasured confounding on estimates of vaccine impact [7, 11], and the spatiotemporal model leverages variations in vaccine uptake between mesoregions to estimate vaccine-associated changes. We performed sensitivity analyses for both of these analyses that confirmed our findings.

This study also has several important limitations. We relied on a nonspecific definition (all-cause pneumonia) drawn from a large administrative database. As we have previously reported [6], these types of administrative data can be subject to a number of important quality issues that can influence the interpretation of the analyses. The SC approach is designed to minimize biases in these data, but it is impossible to eliminate their influence. In addition, the database we used only captures public healthcare. This covers approximately 80% of the population nationally, but coverage of the system varies by region and over time. Finally, we did not have a way of auditing the quality of the data. In particular, the definition of “all-cause pneumonia” might vary. This would be an issue if the specificity changes (eg, fraction that are X-ray confirmed) over time or between regions. Likewise, we did not have a way to audit the hospitalization [21] or vaccine uptake data on the national level or to evaluate timeliness of vaccination, something that can be done in certain localities using record linkages [22].

In conclusion, we have demonstrated that declines in all-cause pneumonia associated with the introduction of PCV10 are consistent across a broad range of development levels. This suggests that the vaccine could have a significant impact in low-income populations.

## Supplementary Data

Supplementary materials are available at *Clinical Infectious Diseases* online. Consisting of data provided by the authors to benefit the reader, the posted materials are not copyedited and are the sole responsibility of the authors, so questions or comments should be addressed to the corresponding author.

## Supplementary Material

Table_S1

Supplementary_Methods

Supplemental_figures

## Notes

***Financial support.*** This work was funded by the Bill & Melinda Gates Foundation (OPP1114733). This publication was also made possible by the National Institutes of Health (NIH), National Institute of Allergy and Infectious Diseases (R01AI123208 and R56AI110449); NIH/National Institute on Aging (P30AG021342; Scholar at the Claude D. Pepper Older Americans Independence Center at Yale University School of Medicine); and a Clinical and Translational Science Awards grant (UL1 TR001863 and KL2 TR001862) from the National Center for Advancing Translational Science, components of the NIH, and NIH Roadmap for Medical Research.

***Potential conflicts of interest.*** D. M. W. has received an investigator-initiated research grant from Pfizer and consulting fees from Pfizer, Merck, GSK, and Affinivax. L. S. and R. J. T. have an ownership interest in Sage Analytica, a research consultancy with government, nongovernment, and pharmaceutical industry clients, including an investigator-initiated research grant from Pfizer (completed in 2013). All remaining authors: No reported conflicts. All authors have submitted the ICMJE Form for Disclosure of Potential Conflicts of Interest. Conflicts that the editors consider relevant to the content of the manuscript have been disclosed.

## References

[1] Feikin DR, Kagucia EW, Loo JD; Serotype Replacement Study Group Serotype-specific changes in invasive pneumococcal disease after pneumococcal conjugate vaccine introduction: a pooled analysis of multiple surveillance sites. PLoS Med 2013; 10:e1001517. 24086113 10.1371/journal.pmed.1001517 PMC3782411

[2] Loo JD, Conklin L, Fleming-Dutra KE Systematic review of the effect of pneumococcal conjugate vaccine dosing schedules on prevention of pneumonia. Pediatr Infect Dis J 2014; 33(Suppl 2):S140–51. 24336056 10.1097/INF.0000000000000082 PMC3944478

[3] Afonso ET, Minamisava R, Bierrenbach AL Effect of 10-valent pneumococcal vaccine on pneumonia among children, Brazil. Emerg Infect Dis 2013; 19:589–97. 23628462 10.3201/eid1904.121198 PMC3647414

[4] Control CfD, Prevention. Progress in introduction of pneumococcal conjugate vaccine—worldwide, 2000–2008. MMWR Morb Mortal Wkly Rep 2008; 57: 1148. 18946462

[5] Johnson HL, Deloria-Knoll M, Levine OS Systematic evaluation of serotypes causing invasive pneumococcal disease among children under five: the pneumococcal global serotype project. PLoS Med 2010; 7: e1000348. 20957191 10.1371/journal.pmed.1000348 PMC2950132

[6] Schuck-Paim C, Taylor RJ, Simonsen L Challenges to estimating vaccine impact using hospitalization data. Vaccine 2017; 35:118–24. 27899227 10.1016/j.vaccine.2016.11.030 PMC5664940

[7] Bruhn CA, Hetterich S, Schuck-Paim C Estimating the population-level impact of vaccines using synthetic controls. Proc Natl Acad Sci U S A 2017; 114:1524–9. 28154145 10.1073/pnas.1612833114 PMC5321019

[8] Programme UND. Human Development Atlas in Brazil Available at: http://www.atlasbrasil.org.br/2013/en/.

[9] Health BMo. National Immunization Program Information System (PNI) Available at: http://pni.datasus.gov.br/.

[10] Schuck-Paim C, Taylor R, Lindley D, Klugman KP, Simonsen L Use of near-real-time medical claims data to generate timely vaccine coverage estimates in the US: the dynamics of PCV13 vaccine uptake. Vaccine 2013; 31:5983–8. 24144470 10.1016/j.vaccine.2013.10.038

[11] Brodersen KH, Gallusser F, Koehler J, Remy N, Scott SL Inferring causal impact using Bayesian structural time-series models. Ann Appl Stat 2015; 9: 247–74.

[12] Gelfand AE, Kim H-J, Sirmans C, Banerjee S Spatial modeling with spatially varying coefficient processes. J Am Stat Assoc 2003; 98: 387–96. 10.1198/016214503000170 PMC11484471 39421645

[13] Besag J, York J, Mollie A Bayesian image restoration with two applications in spatial statistics (with discussion). Ann Inst Stat Math 1991; 43: 1–59.

[14] Martins TG, Simpson D, Lindgren F, Rue H Bayesian computing with INLA: new features. Comput Stat Data Anal 2013; 67: 68–83.

[15] Rue H, Martino S, Chopin N Approximate Bayesian inference for latent Gaussian models by using integrated nested Laplace approximations. J R Stat Soc Series B Stat Methodol 2009; 71: 319–92.

[16] Team RC. R: A language and environment for statistical computing. Vienna, Austria: R Foundation for Statistical Computing 2013 2014.

[17] Gatera M, Uwimana J, Manzi E Use of administrative records to assess pneumococcal conjugate vaccine impact on pediatric meningitis and pneumonia hospitalizations in Rwanda. Vaccine 2016; 34:5321–8. 27639280 10.1016/j.vaccine.2016.08.084

[18] Mackenzie GA, Hill PC, Sahito SM Impact of the introduction of pneumococcal conjugate vaccination on pneumonia in The Gambia: population-based surveillance and case-control studies. Lancet Infect Dis 2017. 10.1016/S1473-3099(17)30321-3 PMC5589209 28601421

[19] Weinberger DM Filling evidence gaps on the impact of pneumococcal vaccines. Lancet Infect Dis 2017. doi: 10.1016/S1473-3099(17)30328-6. 10.1016/S1473-3099(17)30328-6 PMC6005207 28601420

[20] Walker CL, Rudan I, Liu L Global burden of childhood pneumonia and diarrhoea. Lancet 2013; 381:1405–16. 23582727 10.1016/S0140-6736(13)60222-6 PMC7159282

[21] Sgambatti S, Minamisava R, Afonso ET, Toscano CM, Bierrenbach AL, Andrade AL Appropriateness of administrative data for vaccine impact evaluation: the case of pneumonia hospitalizations and pneumococcal vaccine in Brazil. Epidemiol Infect 2015; 143:334–42. 24759601 10.1017/S0950268814000922 PMC9206775

[22] Sartori AL, Minamisava R, Afonso ET Timeliness and risk factors associated with delay for pneumococcal conjugate 10-valent routine immunization in Brazilian children. Vaccine 2017; 35:1030–6. 28108230 10.1016/j.vaccine.2017.01.012

