# Supplementary material for: Impact of Pneumococcal Conjugate Vaccines on Pneumonia Hospitalizations in High- and Low-Income Subpopulations in Brazil
Source: Clin Infect Dis. 2017 Jul 22;65(11):1813–8. doi: 10.1093/cid/cix638 (PMC5848248; doi:10.1093/cid/cix638)
Supplement: Table_S1 [file cix638_suppl_table_s1.pdf]

HDI	Age Group	All covariates	Drop top weighted covariate	Drop 2 top weighted covariates	Drop 3 top weighted covariates
		Rate ratio (95%CI)	Rate ratio (95%CI)	Rate ratio (95%CI)	Rate ratio (95%CI)
Low/ very low	<12m	0.6717 (0.6017,0.7354)	0.6035 (0.4767,0.741)	0.6955 (0.6308,0.7565)	0.7005 (0.6391,0.7612)
Medium	<12m	0.7517 (0.6926,0.8136)	0.7467 (0.6802,0.8228)	0.7478 (0.6834,0.8337)	0.7528 (0.6893,0.8362)
High/very high	<12m	0.7334 (0.6498,0.8515)	0.8335 (0.701,0.9776)	0.8247 (0.6939,0.9661)	0.8166 (0.696,0.9507)
All HDI combined	<12m	0.7464 (0.6814,0.8183)	0.7809 (0.6768,0.9114)	0.8038 (0.7064,0.9238)	0.7895 (0.6965,0.8914)
Low/ very low	12-23m	0.6782 (0.619,0.7367)	0.6899 (0.6346,0.7677)	0.7095 (0.6373,0.7809)	0.7072 (0.6331,0.7849)
Medium	12-23m	0.8093 (0.7441,0.8797)	0.7378 (0.6705,0.8023)	0.73 (0.6616,0.8065)	0.7091 (0.656,0.7799)
High/very high	12-23m	0.7676 (0.6975,0.8476)	0.8148 (0.7285,0.8966)	0.7759 (0.6864,0.8741)	0.7685 (0.6824,0.8735)
All HDI combined	12-23m	0.7595 (0.7004,0.8291)	0.785 (0.6974,0.8723)	0.7664 (0.6778,0.8799)	0.744 (0.6733,0.8274)
Low/ very low	2-4y	0.7213 (0.6624,0.7825)	0.7246 (0.6645,0.7913)	0.7429 (0.6818,0.8029)	0.7573 (0.7022,0.8078)
Medium	2-4y	0.7521 (0.6897,0.8287)	0.7556 (0.6982,0.8295)	0.7603 (0.7109,0.8315)	0.7549 (0.7091,0.8116)
High/very high	2-4y	0.8597 (0.7488,0.9705)	0.81 (0.7111,0.901)	0.7804 (0.6868,0.8882)	0.7789 (0.684,0.8871)
All HDI combined	2-4y	0.7744 (0.694,0.8572)	0.782 (0.7009,0.8606)	0.7614 (0.6873,0.8524)	0.7587 (0.6856,0.8533)
Low/ very low	5-17y	0.7243 (0.6159,0.8113)	0.6739 (0.5829,0.7878)	0.6726 (0.5853,0.7749)	0.6893 (0.5943,0.849)
Medium	5-17y	0.7897 (0.6981,0.9022)	0.7334 (0.639,0.8268)	0.7531 (0.6556,0.8595)	0.7544 (0.6535,0.8596)
High/very high	5-17y	0.9289 (0.762,1.0657)	0.7841 (0.617,0.9507)	0.7163 (0.5949,0.8297)	0.7494 (0.6597,0.8457)
All HDI combined	5-17y	0.8073 (0.7082,0.9177)	0.7424 (0.6409,0.8603)	0.7008 (0.6018,0.8169)	0.7437 (0.6343,0.8533)
Low/ very low	18-39y	0.857 (0.7314,0.9752)	0.9152 (0.7819,1.0502)	0.8777 (0.7742,0.9758)	0.861 (0.7216,1.0358)
Medium	18-39y	0.8088 (0.7067,0.9397)	0.7076 (0.6176,0.817)	0.7278 (0.6377,0.8439)	0.7585 (0.6469,0.906)
High/very high	18-39y	0.8704 (0.7435,0.9772)	0.6646 (0.5672,0.7631)	0.7086 (0.5943,0.8031)	0.6722 (0.5492,0.8172)
All HDI combined	18-39y	0.8 (0.6917,0.9142)	0.6428 (0.5605,0.7339)	0.7276 (0.6038,0.8527)	0.7661 (0.6547,0.8671)
Low/ very low	40-64y	0.8283 (0.6596,0.9596)	0.7189 (0.61,0.8762)	0.8393 (0.7182,0.9802)	0.8629 (0.7358,0.9961)
Medium	40-64y	0.8677 (0.7179,1.0244)	0.8378 (0.7055,1.0033)	0.8845 (0.7443,1.0386)	0.848 (0.7341,0.9639)
High/very high	40-64y	0.9648 (0.856,1.0837)	0.8511 (0.741,0.9753)	0.8675 (0.7548,0.9986)	0.8603 (0.7508,0.9978)
All HDI combined	40-64y	0.941 (0.7906,1.101)	0.8392 (0.7212,0.9818)	0.8557 (0.7184,1.0263)	0.8951 (0.7448,1.0551)
Low/ very low	65-79y	0.8571 (0.7297,1.0131)	0.8906 (0.72,1.0549)	0.8883 (0.7109,1.072)	0.9843 (0.8325,1.1013)
Medium	65-79y	0.9739 (0.8388,1.1073)	0.9376 (0.8196,1.0821)	0.9088 (0.7875,1.0609)	0.9525 (0.8481,1.101)
High/very high	65-79y	0.9228 (0.8243,1.0348)	0.9492 (0.8527,1.0551)	0.9584 (0.858,1.0717)	0.9618 (0.8521,1.0773)
All HDI combined	65-79y	0.9475 (0.8112,1.0979)	0.9031 (0.7908,1.0697)	0.942 (0.7834,1.1671)	0.9588 (0.788,1.1779)
Low/ very low	80+y	1.0453 (0.9117,1.1754)	0.9606 (0.8345,1.1004)	0.9845 (0.8534,1.1165)	0.9453 (0.813,1.0846)
Medium	80+y	0.9506 (0.8212,1.0943)	1.007 (0.8971,1.1206)	0.9953 (0.8836,1.1222)	1.007 (0.8926,1.1366)
High/very high	80+y	1.0054 (0.9108,1.1158)	1.0073 (0.9007,1.1412)	1.0409 (0.9144,1.1743)	1.018 (0.8968,1.1671)
All HDI combined	80+y	0.9713 (0.8528,1.0996)	1.008 (0.8642,1.1455)	0.9874 (0.8481,1.1322)	0.9984 (0.8454,1.155)
