# Supplementary material for: Impact of Pneumococcal Conjugate Vaccines on Pneumonia Hospitalizations in High- and Low-Income Subpopulations in Brazil
Source: Clin Infect Dis. 2017 Jul 22;65(11):1813–8. doi: 10.1093/cid/cix638 (PMC5848248; doi:10.1093/cid/cix638)
Supplement: Supplementary_Methods [file cix638_suppl_supplementary_methods.docx]

**SUPPLEMENTARY METHODS**

*Details on the spatiotemporal model*

We model the counts of pneumonia hospitalizations at meso-region *s* during year *t* using Poisson regression such that $Y\left( s,t \right)|\lambda\left( s,t \right)\sim\mathrm{Poisson}\left\{ \lambda\left( s,t \right) \right\}$ where $Y\left( s,t \right)$ is the conditionally independent observed count and $\lambda\left( s,t \right)$ represents the mean/variance of the Poisson distribution. We allowed each meso-region to potentially have a unique association with vaccine uptake over time through use of varying slopes in order to see how the vaccine association changes across Brazil spatially as well as in terms of development level. The slope in a given meso-region was modeled as a function of its development level and its region, and a meso-region-specific random effect that captured excess variability unexplained by development and region. Each meso-region also had its own intercept, which captured baseline differences in the occurrence of all-cause pneumonia hospitalizations prior to the introduction of PCV10. To control for spatiotemporal variation in the counts, we also include additional control time series at the meso-region and region levels such as all-cause hospitalizations (all age groups), and the top-weighted variables identified in the SC analysis.

The expected count is modeled as a function of spatially- and temporally-varying covariates, random effects, and PCV uptake such that $\ln\left\{ \lambda\left( s,t \right) \right\}=\mathbf{x}\left( s,t \right)^{T}\boldsymbol{\gamma}+\tilde{\beta}_{0}\left( s \right)+w\left( s,t \right)\tilde{\beta}_{1}\left( s \right)+\theta\left( s,t \right)$ where $\mathbf{x}\left( s,t \right)$ is a vector of spatially- and temporally-varying covariates unique to meso-region *s* at time *t*; $\boldsymbol{\gamma}$ is a vector of unknown regression coefficients relating the covariates to the response; $\theta\left( s,t \right)$ represents the observation-level random effect that accounts for potential overdispersion in the Poisson data; $\tilde{\beta}_{0}\left( s \right)$ and $\tilde{\beta}_{1}\left( s \right)$ represent meso-region-specific intercept and slope parameters, respectively; and $w\left( s,t \right)$ represents the transformed vaccine uptake amount in meso-region *s* at time *t*.

The vector of covariates include yearly indicators to control for global changes in pneumonia hospitalizations across Brazil through time, all cause hospitalization counts at the meso-region level and aggregated to the region level, as well as potential confounding variables (explained in Methods section), also at the region and meso-region levels. All of the count variables are modeled on the log + 0.50 scale and standardized for computational stability during modeling.

In addition to the covariates mentioned in the main text, we included the top-weighted covariates for each age group identified with the SC model. These included: bronchitis/bronchiolitis [J20-22] (age groups < 12 months, 12-23 months, 2-5 years, and 40-65 years), malnutrition [E40-46] (age groups < 12 months, 12-23 months, and 2-5 years), diseases of the digestive systems [K00-99] (2-5 years), diseases of the genitourinary system [N00-99] (5-17 years), diseases of the circulatory system [I00-99] (18-39 years), and diseases of the eye and adnexa [H00-99] (40-65 years). The control variables were selected separately for each age group based on the posterior weighting observed when using the SC method. We selected the 1-2 highest weighted control variables from the SC analysis, and included all-cause hospitalization counts for all age groups.

The observation-level random effects are assumed to be independent and identically normally distributed, centered at zero with a common variance parameter. The PCV uptake proportion is centered at each year in order to break the temporal correlation with time, as PCV uptake is increasing consistently across time across all meso-regions. To accomplish this centering, we calculated the mean coverage for the year across all meso-regions and subtracted this from the meso-region-specific estimates. We also add a constant to the post-vaccine years to ensure that the centered uptake is strictly positive across all years. This is a requirement of using the spatially varying coefficient model as detailed in past work [21]. Uptake amounts before the introduction of the vaccine are fixed at zero. This transformation allows us to isolate the impact of PCV without confusing it with global changes across time. For example, if each meso-region had the exact same uptake at each year (possibly changing in each year), the model would attribute all changes in hospitalizations to the yearly indicators instead of the vaccine association. Without centering, it would be difficult to determine what percent of the change over time is due to variations in vaccine uptake between meso-region and what percent is due to secular changes occurring across Brazil. In this way, we are careful not to incorrectly attribute secular trends in pneumonia hospitalizations to the vaccine.

The intercepts and slopes are allowed to vary by meso-region. Additionally, we incorporate the development and region information for a specific meso-region when estimating these parameters. This allows us to directly assess whether the vaccine association or baseline risk of pneumonia hospitalizations differ by these covariates. We also include spatially correlated random effects that account for the possibility that slopes/intercepts in close proximity may be more similar, even after controlling for other spatially varying factors. The model for the intercepts and slopes are given as $\tilde{\beta}_{0}\left( s \right)=\beta_{0}+\sum_{j=2}^{3} I\{z\left( s \right)\in C_{j}\}\delta_{0j}+\sum_{j=2}^{5} I\left\{ r\left( s \right)=j \right\}\delta_{1j}+\beta_{0}(s)$ and $\tilde{\beta}_{1}\left( s \right)=\beta_{1}+\sum_{j=2}^{3} I\{z\left( s \right)\in C_{j}\}\lambda_{0j}+\sum_{j=2}^{5} I\left\{ r\left( s \right)=j \right\}\lambda_{1j}+\beta_{1}\left( s \right)$ respectively, where $I(.)$ is an indicator function that is equal to one if the input statement is true and is equal to zero otherwise; $z\left( s \right)$ is the development level of meso-region *s*; *C_j_* represents a particular level of development (j=1: Low, j=2: Medium, j=3: High/Very High); $r\left( s \right)$ is the geographic region containing meso-region *s* (1: North, 2: Northeast, 3: Southeast, 4: South, 5: Central West); and $\delta_{0j}$, $\delta_{1j}$, $\lambda_{0j}$, $\lambda_{1j}$ are unknown regression parameters that describe the changes in the intercept and slope parameters at different levels of development and at different regions. The spatially varying random effects ($\beta_{0}(s)$, $\beta_{1}\left( s \right)$) are assumed to be independent of each other and follow the Besag, York, Mollie model for spatial random effects[22] that partitions the single random effect into a piece that accounts for spatial correlation through use on an intrinsic conditional autoregressive model, and a piece that is spatially independent to account for excess non-spatial variability in the effects. This model ensures that we do not spatially over- smooth the parameter estimates. This could occur if we attributed all of the excess variability towards spatially correlated error, ignoring the possible non-spatial variability.

A flat prior was assigned for the overall intercept term, $\beta_{0}$, with independent vague normal prior distributions centered at 0 with a variance of 1,000 used for the remaining covariate regression parameters ($\gamma_{j},\beta_{1},\delta_{0k},\lambda_{0k},\delta_{1l},\lambda_{1l};j=1,\ldots p;k=2,3;l=2,\ldots,5$). The variance parameters controlling the spatial random effects are assigned independent, weakly informative Inverse Gamma(1, 0.0005) prior distributions. The variance parameter for the overdispersion random effect is given a weakly informative Inverse Gamma(1, 0.00005) prior distribution. These weakly informative prior choices represent the default settings used by R-INLA and represent our lack of prior information regarding the true parameter values. In subsequent sensitivity analyses, we found that these prior choices did not have a noticeable effect on the resulting posterior inference.

*Marginal posterior mean estimation*

In order to summarize the meso-region vaccine effect output from the spatiotemporal model, we calculate marginal posterior mean estimates along with 95% credible intervals. Marginal posterior means allow us to quantify the overall effect of PCV10 uptake across all meso-regions by correctly accounting for the balance of development and region seen across all of the meso-regions, giving higher weight to parameters from categories that are more commonly seen in the Brazilian data. The overall vaccine effect marginal mean is defined as $\beta_{1}+\sum_{j=2}^{3} p_{0j}\lambda_{0j}+\sum_{j=2}^{5} p_{1j}\lambda_{1j}$ where $p_{0j}$ is the proportion of meso-regions in development category *j* and $p_{1j}$ is the proportion of meso-regions in region *j*. These marginal means are then exponentiated to produce RR estimates.

*Sensitivity analysis for the spatiotemporal model*

The random swapping technique was repeated 50 times for each age group. The swapping was restricted to meso-regions from the same region in order to avoid breaking the associations between spatially varying confounders that may be difficult to differentiate from vaccine uptake, as we generally expect vaccine uptake to be similar across meso-regions that are spatially similar (though not identical). After fitting the spatiotemporal model to the 50 randomly generated datasets, we calculated the average vaccine effect for each meso-region and compare this estimate with the observed estimate from the true analysis. In order to compare the estimates, we subtracted the swapped vaccine effect estimate from the true vaccine effect estimate for each meso-region, exponentiated to convert to the RR scale, and plotted them as in the main analysis.
