# Supplementary material for: Impact of Pneumococcal Conjugate Vaccines on Pneumonia Hospitalizations in High- and Low-Income Subpopulations in Brazil
Source: Clin Infect Dis. 2017 Jul 22;65(11):1813–8. doi: 10.1093/cid/cix638 (PMC5848248; doi:10.1093/cid/cix638)

**Figure S1:** Figure S1: A) Histogram of the range of human development index (HDI) values for the municipalities of Brazil or globally. B) Map of HDI by municipality in Brazil. C) Uptake of PCV10 by HDI level, with each dot representing a meso-region.

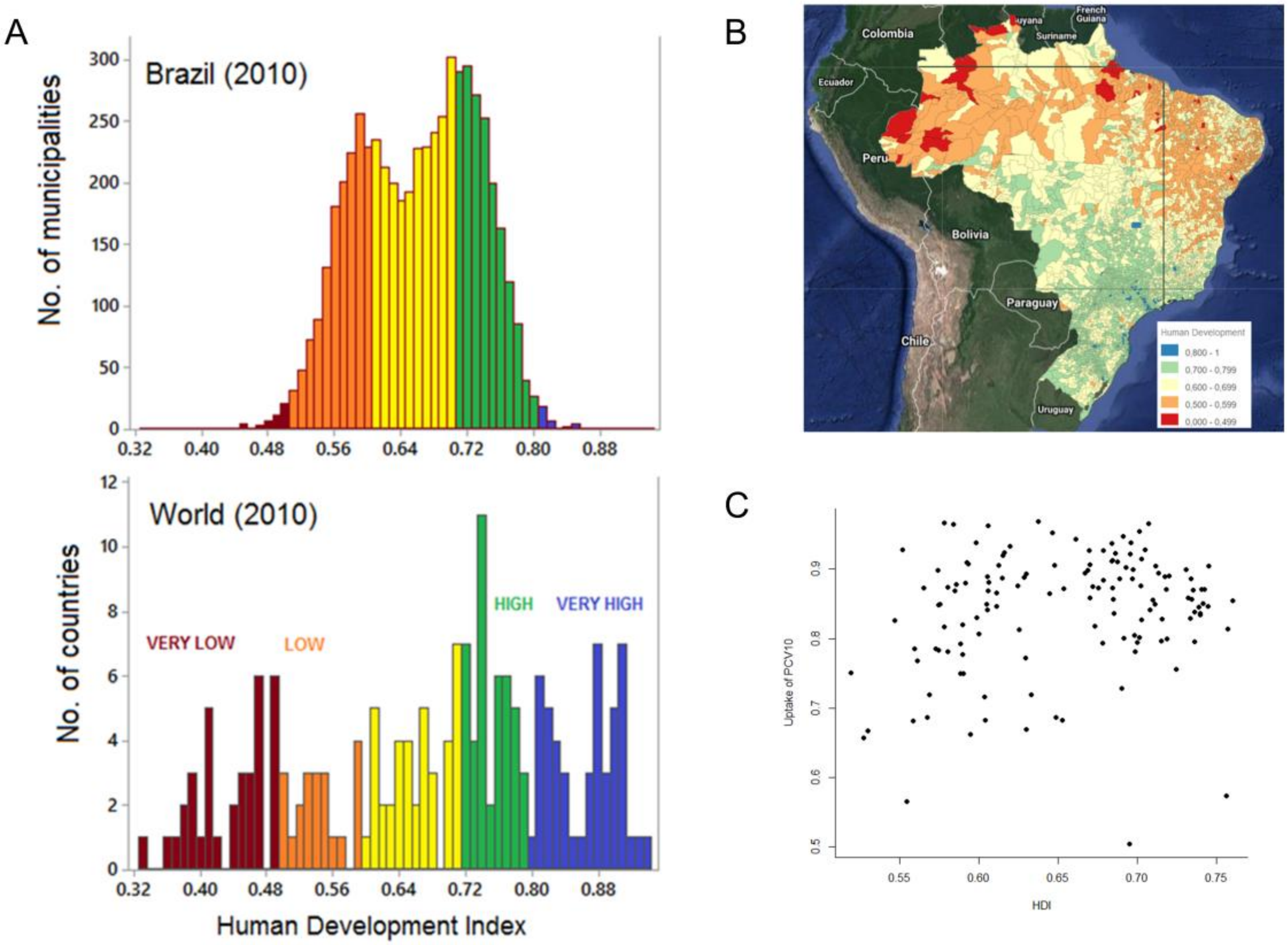

**Figure S2:** Changes in vaccine uptake and volume of hospitalizations by year and human development index level. **A.** Vaccine uptake by mesoregion and year, colored by HDI group. B. All-cause pneumonia hospitalizations by year and HDI group. C. All non-respiratory hospitalizations by year and HDI group. Red= Low/very low HDI; blue=medium HDI; green=High/very high HDI

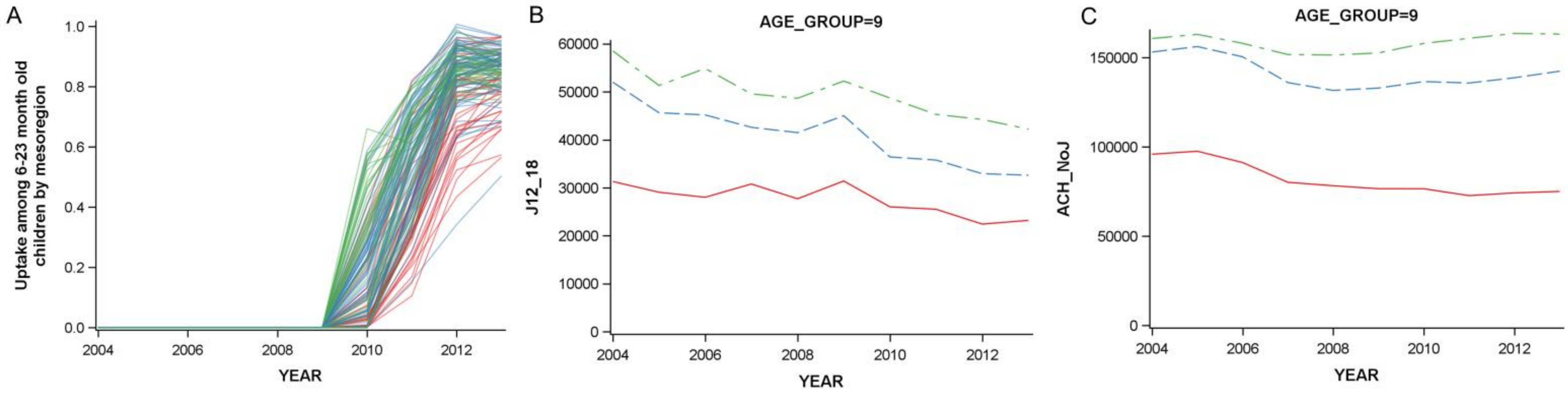

**Figure S3:** Time series plots comparing the observed and counterfactual number of pneumonia cases in each month. The black line shows the observed cases, and the white line and grey shaded area shows the median and 95% credible interval for the counterfactual estimated form the synthetic control model. The plot is stratified by human development level (HDI).

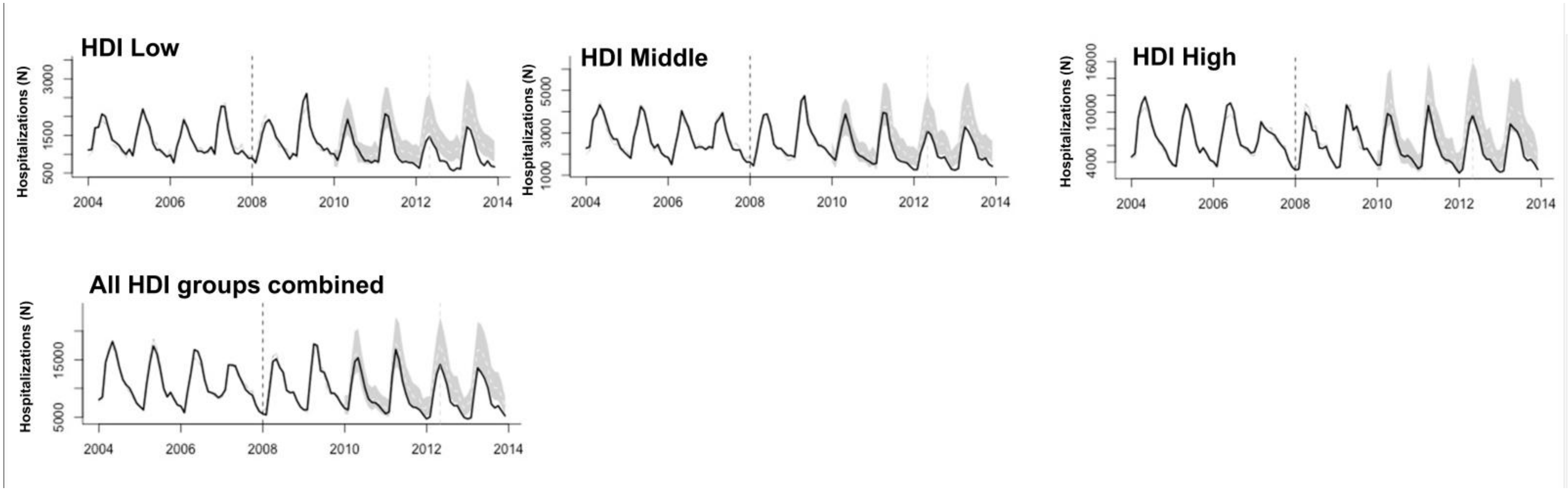

**Figure S4**: Change in pneumonia hospitalizations following the introduction of PCV10 in Brazil as estimated with A) the null model that only adjust for non-respiratory hospitalizations and seasonality and B) the synthetic control model, comparing the observed and predicted number of cases in 2012-2013; by age group and development level. Median +/-95% credible intervals.

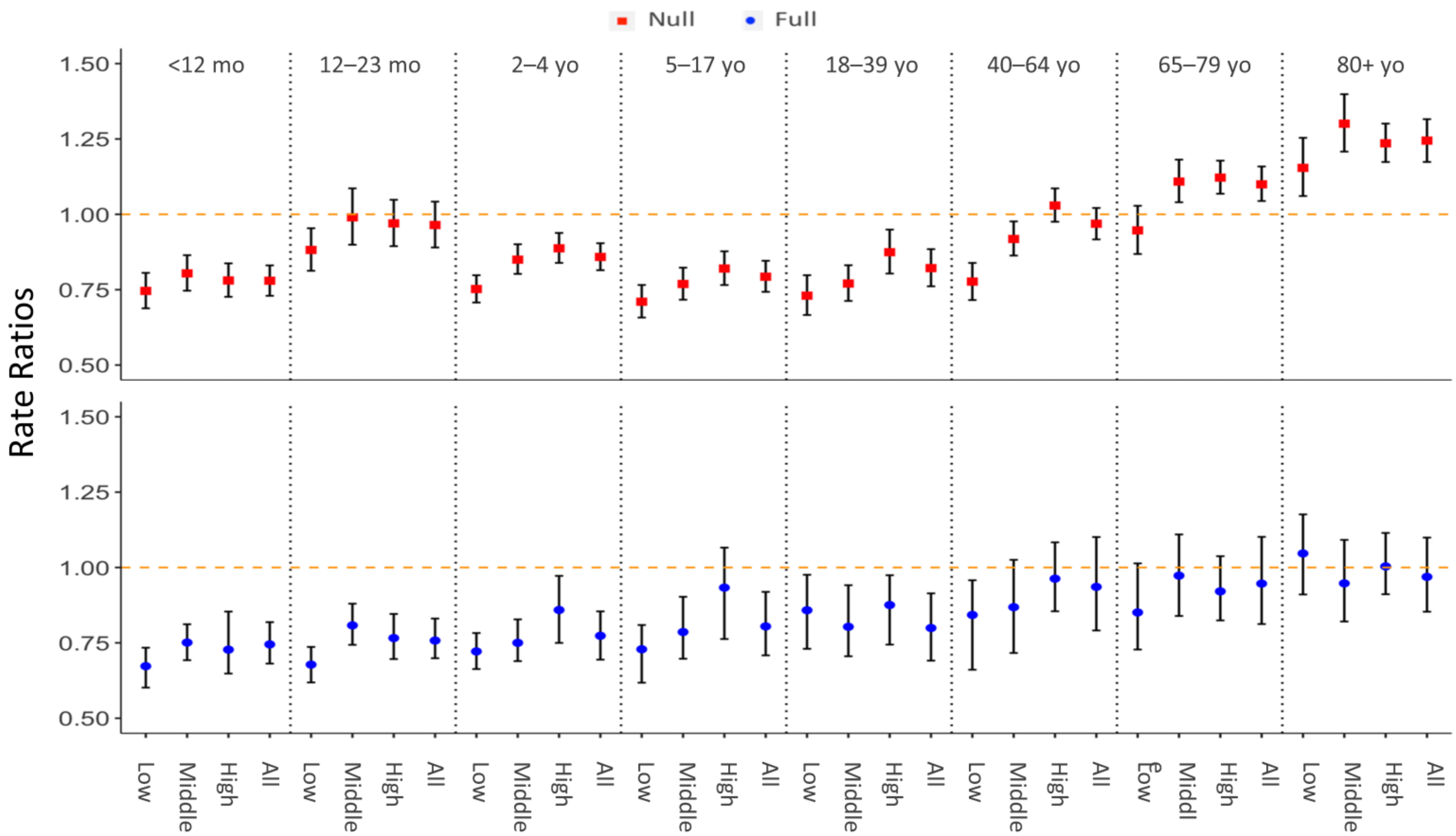

**Figure S5**: Rate ratio by meso-region, ordered by region. The size of the bubble is proportional to the number of cases of pneumonia in that age group and meso-region in 2013. Colors denote regions: red=North, blue=Northeast, green=Southeast, purple=South, orange=Center-West.

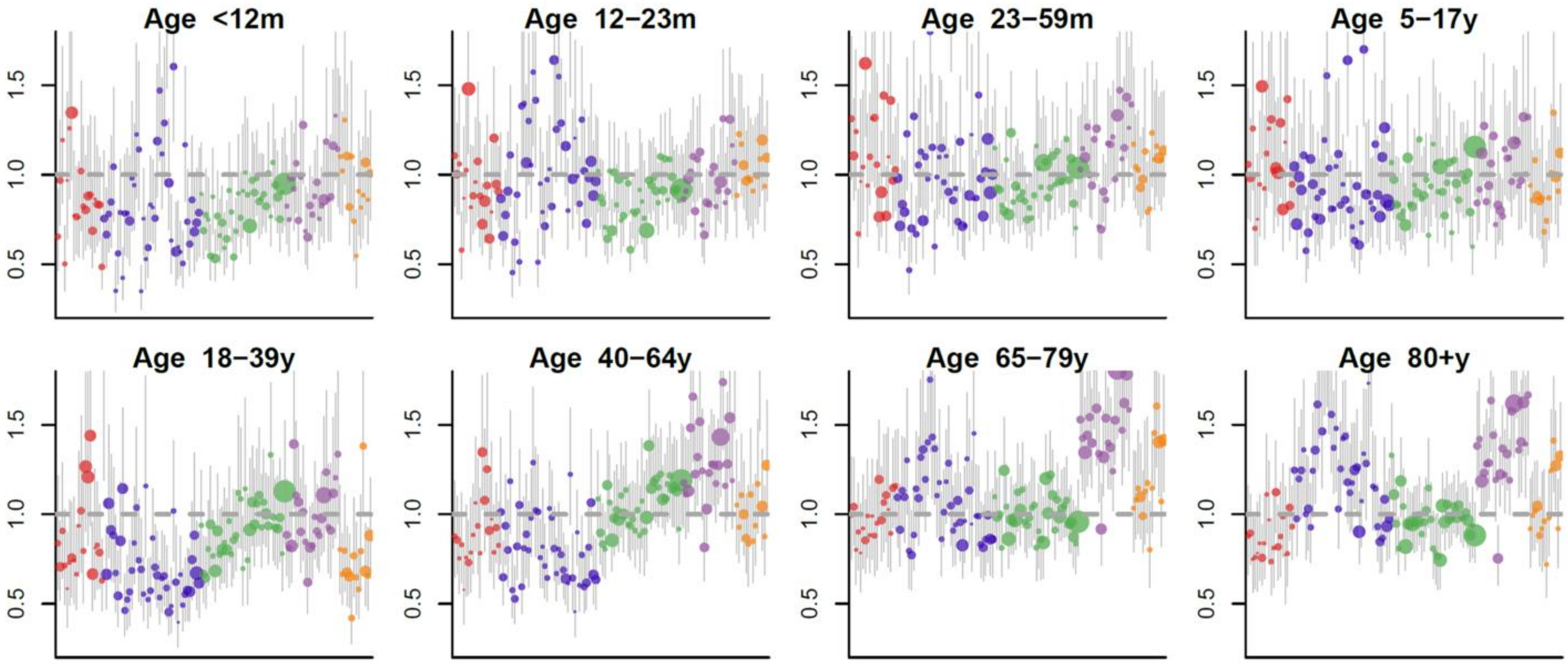

Supplement: Supplemental_figures [file cix638_suppl_supplemental_figures.pdf]
